# Current status of soil transmitted helminths and *Schistosoma mansoni* infection among children in two primary schools in North Gondar, Northwest Ethiopia: a cross sectional study

**DOI:** 10.1186/1756-0500-7-88

**Published:** 2014-02-10

**Authors:** Biniam Mathewos, Abebe Alemu, Desalegn Woldeyohannes, Agersew Alemu, Zelalem Addis, Moges Tiruneh, Mulugeta Aimero, Afework Kassu

**Affiliations:** 1 Department of Immunology and Molecular Biology, School of Biomedical and Laboratory Sciences, College of Medicine and Health Sciences, University of Gondar, Gondar, Ethiopia; 2 Department of Medical Parasitology, School of Biomedical and Laboratory Sciences, College of Medicine and Health Sciences, University of Gondar, Gondar, Ethiopia; 3 Department of Medical Microbiology, School of Biomedical and Laboratory Sciences, College of Medicine and Health Sciences, University of Gondar, Gondar, Ethiopia; 4 Department of Biology, Faculty of Natural and Computational Sciences, University of Gondar, Gondar, Ethiopia; 5 Department of Public Health, School of Medical and Health Sciences, Addis Ababa Science and Technology University, Addis Ababa, Ethiopia

**Keywords:** Soil transmitted helminths, Schistosoma mansoni, School children

## Abstract

**Background:**

School age children are one of the groups at high risk for intestinal parasitic infections especially in developing countries like Ethiopia as the supply of good quality drinking water and latrine coverage are poor. Though there are previous data on the prevalence of soil transmitted helminths (STHs) and *Schistosoma mansoni* infection among these high risk groups current status in the study area is unknown. Therefore, the aim of this study was to determine the current prevalence and associated risk factors of STHs and *S. mansoni* infections among school children.

**Methods:**

A cross-sectional study was carried out in Gorgora and Chuahit towns, North Gondar Zone, North West Ethiopia from January 20 to February 25, 2012 involving 261 school children. A pre-tested and structured questionnaire was used to collect socio-demographic data and possible risk factors. Stool samples were collected and examined for intestinal parasites using Kato Katz method. Chi-square test was used to see if there is association between sociodemographic factors and other risk factors for STH and *S. mansoni* infection and odds ratio with 95% CI was computed as measures of association. P < 0.05 was taken as statistically significant.

**Results:**

Out of the 261 study participants, 174 (66.7%) were infected with one or more species of intestinal parasites. *Ascaris lumbricoides* was the predominant isolates (39.8%) followed by *Trichuris trichiura* (6.1%) and Hookworms (4.9%). *Schistosoma mansoni* was detected in 33.7% of the children. Among infected individuals, 9.5% were coinfected by *S. mansoni* and *A. lumbricoides* and 1.5% with *S. mansoni* and *T. trichiura.* Swimming habit (OR: 2.536, 95% CI: 1.122, 5.737, P = 0.022) was significantly associated with *S. mansoni* infection*.*

**Conclusion:**

The prevalence of STH and *S. mansoni* was high among school children. This should call for implementation of an integrated strategy to reduce morbidity and control of transmission of STH and *S. mansoni.*

## Background

Soil transmitted helminthiasis and schistosomiasis are widely distributed and among the major medical and public health problems in many parts of the world. World Health Organization (WHO) estimated that 1.45 billion people are infected with *A. lumbricoids*, 1.3 billion with Hookworms and 1.05 billion with *T. trichiura*[1]. Schistosomiasis is also common in many parts of the world and about 200 million people are infected globally [2]. It is endemic in 76 countries and also it is one of a public health concern in developing countries. Approximately 80% of the 200 million people infected world-wide live in sub-Saharan Africa where *S. mansoni* is widespread [3,4].

Ethiopia is a country with the lowest quality drinking water supply and latrine coverage in the world [5]. Hence, this unsafe and inadequate provision of water together with unhygienic living conditions and unsanitary waste management allow intestinal parasites and other communicable diseases to flourish in various localities [6]. As a result, intestinal parasites are common in most parts of Ethiopia and helminthic infections are the second most predominant causes of outpatient morbidity in the country. Among all of the helminthic infections, Ascariasis is the most prevalent in Ethiopia [7]. Schitosomiasis is also common in the area and if we see the distribution among the different parts of Ethiopia, it is a common disease in the northern region as compared to south and south west regions of Ethiopia [8].

Age is one of a risk factor for intestinal parasitic infection. Children who reach school age are at high risk for intestinal parasitic infections. Poor hygiene, low immune status, overcrowding, close contact with soil and to each other, lack of latrine, and low provision of water in schools are some of the factors that put school age children at high risk for intestinal parasitic infection [9].

Intestinal parasitic infections have known negative effects on the growth, appetite, and cognitive performance of school age children. Besides, the physical wellbeing and the active participation of school children will be compromised by these infections [10-13]. WHO emphasizes that appropriate treatment can reduce the burdon on majority of these children [14].

STHs and Schistosoma infections can be reduced based on interventions like regular anti-helminthic treatment, improved water supply, sanitation and health education [15]. STH infections have not been targeted for control in Ethiopia [16], though mass deworming as a component of the enhanced outreach strategy targeting under five children started in 2004 [17]. High coverage and low cost delivery of anti helminthic treatment has been achieved in some settings but improvement of sanitation is more complex since access to improved sanitation is very low in countries like Ethiopia [18,19].

Prevalence of intestinal helminths has been studied in different parts of Ethiopia [20-26]. However, the current prevalence of STH and schistosomiasis was not well addressed in different parts of Ethiopia including our study area. Therefore, the aim of this study was to determine the prevalence and associated risk factors of STHs and *S. mansoni* infection among primary school children of Chuahit and Gorgora towns, North Gondar, North West Ethiopia.

## Methods

### Study design and area

Cross-sectional study was conducted from January 20 to February 25, 2012 among primary school children of Gorgora and Chuahit towns of Dembia District, North Gondar Zone, northwest Ethiopia. Gorgora is a small but flourishing roadside town on the shore of Lake Tana (altitude 1800 m), 65 kms South of Gondar town. Chuahit is located to the north of Gorgora. The estimated total population in the two towns is estimated to be 2446 and 6,814 for Gorgora and Chuahit respectively [27]. The average temperature and humidity registered in both towns is 28°C and 22% respectively. Both of the towns have their own health centers that provide primary health care services including family health service, prevention and control of communicable diseases (malaria, HIV, tuberculosis, intestinal parasitic infections) and hygiene and environmental health. And both health centers also give diagnostic and treatment services for major infectious diseases of the area.

### Sample size and sampling technique

The sample size was determined by using single proportion formula at 95% confidence interval (CI) level (Z (1-ά/2) = 1.96). A prevalence of 67% was taken from similar study conducted before sixteen years ago in the same area [28]. A 5% of marginal error was also taken. Therefore, sample size was calculated as n = [Z 1- α/2] 2 P (1-p]/d2, where: n is sample size, P is prevalence of parasites, Z 1-α/2 is CI of 95%, d is marginal error to be tolerated [29]. By this calculation we obtained 340 to be the sample size. Since the total population in the two primary schools was less than 10,000 adjustment was made according to the formula; nf = ni/(1 + ni/N) and a sample size of 254 was found. We added 10% for compensation of non-respondents and the final calculated sample size became 279 among whom 261 had participated in the study.

To determine the proportion of students to be selected from each of the two school and each sections, the sample size which was determined earlier was used. Each school had 10 sections therefore the total number of section was twenty. By dividing our sample size which was 261 by the number of total sections (20 sections), we obtained 13 study participants per one section. Therefore in each section 13 students and 14 from the last section has been taken by using simple random sampling method. To do this we used a table of random numbers to select study participants. As a sampling frame we used a registration list obtained from the schools.

### Data collection and processing

#### Socio demography and risk factor assessment

The study subjects were interviewed to assess whether they fulfill the already prepared inclusion criteria. The inclusion criteria that we used mentioned that those students whose parents or guardians were willing to give written consent and the students not taking anti helminthic drugs for the last 15 days were allowed to be included in this study.

Data on sociodemographic factors and other risk factors for STH and *S. mansoni* infection were collected using a structured questionnaire prepared for the purpose of this study. The questionnaire was prepared in English and then translated to the local language (Amharic) and checked for fitness. A pre-test was conducted on 10% of the school children that are not included in the study and appropriate corrections were made as the results found.

### Parasitological techniques

Sufficient amount of single stool specimen (approximately 5 gm) was collected from each participant using a leak proof, tightly corked container. Samples were prepared using Kato-Katz technique as per the standard [30] and examined using X100 and X400 magnifications. The result was recorded carefully on well prepared format and finally it was attached with the respective questionnaire.

### Quality control

The samples were examined within the 30 minutes of preparation of the sample. All slides were examined twice for confirmation of the result. From all of the slides, 15% were randomly selected and re-examined at the end by experienced laboratory technologist who was blind for the first examination result.

### Data analysis

Data was entered and analyzed using SPSS version 20 computer software after checking its completeness. Data was summarized in percentages and presented in tables. Association between risk factors and parasitological test results was assessed using odds ratio with 95% CI. P-value less than 0.05 were considered statistically significant.

### Ethical consideration

The study was ethically approved by the ethical review committee of the school of biomedical and laboratory sciences, University of Gondar by the letter numbered SBLS/34/2012. The purpose of the study was communicated with Zonal health officers and informed consent was obtained from the parents/guardians of the students. Students infected with *S. mansoni* were treated with 40 mg/kg praziquantel (PZQ) and those infected with STHs were treated with 400 mg albendazole (ALB).

## Results

### Sociodemographic characteristics of the study subjects

Among the 261children (139 males and 122 females), 147 (56.3%) were from the town of Chuahit and 114 (43.7%) from Gorgora. Majority, 224/261(85.8%) study participants were in the age range from 5–15 years and also 157/261 (60.2%) and 251/261(96.2%) were from farmer families and Christians respectively (Table 1).

**Table 1:** Sociodemographic characteristics of children attending Gorgora and Chuahit Elementary Schools, Northwest Ethiopia from January 20 to February 25, 2012

	**Character**	**Frequency**	**Percentage**
Age	<5	8	3.1
	5–15	224	85.8
	>15	29	11.1
Sex	Male	139	53.3
	Female	122	46.7
Religion	Christian	251	96.2
	Muslim	9	3.4
	Others	1	.4
Ethnicity	Amhara	245	93.8
	Tigrei	11	4.2
	Others	5	2
Educational level of the children	Grade 1–4	152	58.2
	Grade 5–8	109	41.8
Address of the participant	Gorgora	147	56.3
	Chuahit	114	43.7
Family occupation	Farmer	157	60.2
	Merchant	45	17.2
	Government employee	59	22.6

### Prevalence of soil transmitted helminthes and schistosoma mansoni

**Figure 1:**
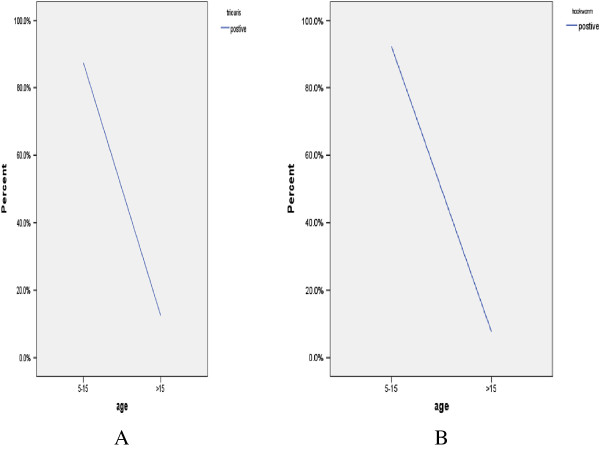
**Prevalence of *T. trichiura *and Hookworms by age. A**. Prevalence of *T. trichiura* by age among children attending Gorgora and Chuahit Elementary Schools, Northwest Ethiopia from January 20 to February 25, 2012. **B**. Prevalence of Hookworms by age among children attending Gorgora and Chuahit Elementary Schools, Northwest Ethiopia from January 20 to February 25, 2012.

**Figure 2:**
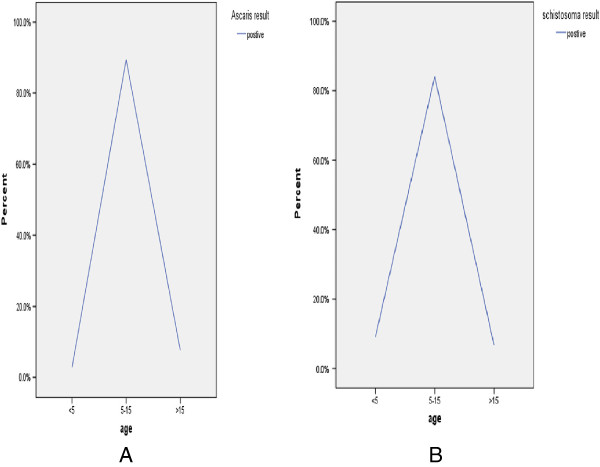
**Prevalence of *A. lumbricoids *and *S. mansoni *by age. A**. Prevalence of *A. lumbricoids* by age among children attending Gorgora and Chuahit Elementary Schools, Northwest Ethiopia from January 20 to February 25, 2012. **B**. Prevalence of *S. mansoni* by age among children attending Gorgora and Chuahit Elementary Schools, Northwest Ethiopia from January 20 to February 25, 2012.

Out of the total study subjects, 174/261 (66.7%) had one or more parasites. *A. lumbricoides* was the predominant isolate (104/261, (39.8%) followed by *Trichuris trichiura* 16/261 (6.1%) and Hookworms 13/261, (4.9%). *S. mansoni* was isolated from 88/261 (33.7%) of the study participants (Table 2). Co-infection rate was higher for *S. mansoni* and *A. lumbricoides* 25/261 (9.5%) followed by *S. mansoni* and *T. trichiura* 4/261 (1.5%). There was a different trends on the prevalence of the parasite among the different age groups in the different parasites (Figures 1 and 2).

**Table 2:** **Prevalence of STH and *S. mansoni *** **in children attending Gorgora and Chuahit Elementary Schools, Northwest Ethiopia from January 20 to February 25, 2012**

**Parasite identified**	**Male (N = 139)**		**Female (N = 122)**		**Total (N = 261)**		**X**^**2**^	**P-value**
	**No%**		**No%**		**No%**			
***S. mansoni***	40	28.7	48	39.3	88	33.7	3.324	0.072
***A. lumbricoides***	57	41	47	38.5	104	39.8	0.167	0.683
***T. trithiura***	7	5	9	7.3	16	6.1	0.619	0.431
***Hookworms***	8	5.7	5	4	13	4.9	0.619	0.431
**Others**	6	4.3	5	4	11	4.2		
**Total**	118	84.8	114	93.4	232	88.8		

### Risk factor analysis for soil transmitted helminthes and *S. mansoni*

Prevalence of *A. lumbricoides* and Hookworms were higher in male study participants while prevalence of *T. trichiura* and *S. mansoni* were higher in the females (Table 2). However there is no statistically significant association between parasite prevalence and being male and female (X^2^ = 0.953, p value =0.335). Swimming habit (OR = 2.536, 95% CI (1.122-5.735) showed statistically significant association with *S. mansoni* infection. However, there was no statistically significant association between other possible risk factors and STH and *S. mansoni* infection (Table 3).

**Table 3:** **Risk factors for both soil transmitted heltminths and *S. mansoni *** **infection in children attending Chuahit and Gorgora Elementary School, Northwest Ethiopia, from January 20 to February 25, 2012**

**Variables**	**N (%)**	**STH and *S. mansoni *** **n%**	**OR (95% CI)**	**P-value**
**Sex**				
Male	139(53.2)	89(64)	0.953( 0.775–1.090)	0.335
Female	122(46.7)	85(69.6)	1	
**Family**				
**Occupation**				
Farmer	157(60)	110(70)	0.581(0.467–1.243)	0.081
Merchant	45(17)	19(42.2)		0.349
Government	59(23)	45(76.3)	0.680(0.576–1. 087)	0.228
Employ			1	
**Address**				
Gorgora	147(56.3)	104(70.7)	1.520(1.005–1.849)	0.113
Chuahit	114(43.6)	70(61.4)	1	

Note: STHs = soil transmitted helminths N = total number of outcomes for each exposure.

N = number of outcome for each exposure.

## Discussion

The overall prevalence of infection with STH and *S. mansoni* among school children of Gorgora and Chuahit towns, 66.7% was very high. This finding is in line with findings of a cross sectional survey conducted on STHs and *S. mansoni* infection among school children in Chilga district, northwest Ethiopia [20]. That study had reported an overall prevalence rate of 68.4%. When specific parasitic prevalence is considered almost similarity was again observed in the prevalence of *A. lumbricoides* in that study (42.9%, range: 22.9%-68.6%) and in the present study (39.8%). However, in other parasites (other than *A. lumbricoides*) differences was observed between the two studies in that the findings of the that study showed a higher prevalence of Hookworms (37.7%, range: 28.0% - 65.5%), *S. mansoni* (19.4%, range: 7.0% - 64.3%) and *T. trichiura* (14.8%, range: 12.7%-20.8%) than the present study which showed a prevalence of 4.9%, 33.7%, and 6.1% for Hookworms, *S. mansoni* and *T. trichiura* respectively. Similarly a study conducted in Dembia plains has shown similar report to the present study with regards to the prevalence of *A. lumbricoides* and *S. mansoni* with infection rates of 41.3%, 35.0%, respectively but difference was observed in prevalence of *T. trichiura* and Hookworms 16.5% and 22.8% respectively [22]. Such differences in the prevalence of the specific parasites may be attributable to the difference in degree of suitableness of the macro and micro environment to the parasites in the different areas.

Higher prevalence in all of STH and *S. mansoni* parasites has been shown in other studies than the present study. For example, study conducted in school children in Adarkay District, northwest Ethiopia [21] showed a prevalence of 55.3% for *S. mansoni*, 43.0% for *A. lumbricoides,* 20.2% for Hookworms and 11.8% for *T. trichiura*. The differences in the prevalence might be due to variation among the two areas in water supply, socioeconomic status, and sanitation. Similarly a study conducted at Zarima town, northwest Ethiopia, showed a higher prevalence in all types of the parasites except for *T. trichiura* than the present study. In that study, overall prevalence of 82.4% was shown and a prevalence of 22%, 19%, 2.5% and 37.9% was reported for *A. lumbricoides,* Hookworms, *T. trichiura* and *S. mansoni* respectively [31]. Some factors like environmental sanitation, personal hygiene, adequateness and quality of water, socioeconomic status, time of the study and climatic factors might be contributing for the difference in prevalence of the different parasites among the two different sites.

When we compare the findings of the present study about prevalence of *S. mansoni* with the study conducted previously in the same area which reported a prevalence of 67% [28], it showed that there is a remarkable decrement in the prevalence of the parasite. The progress in the decrement of the parasites prevalence could be due to different interventions that have been undertaken in the area like deworming and health education. However, the current prevalence cannot be considered to be low since it is still higher than findings of other studies conducted even in areas with higher risk for *S. mansoni* infection. For example in a study conducted at central and southern Tigray, one of the regions of Ethiopia, prevalence of *S. mansoni* was found to be 27% in longstanding irrigated areas, 10.8% in recently constructed irrigation schemes and 1.8% in the non-irrigated rural localities [32]. Similarly in Gondar town and surrounding areas, quite near to our study area, a prevalence of 35.6% was reported [23]. The reasons for higher prevalence of *S. mansoni* in the study area may be due to the fact that the study area is near to Lake Tana which is the biggest lake in the country and also the study area have many small water bodies that can be the sources of transmission for *S. mansoni* even though further extensive study is needed to verify the fact.

The high burden of STH and *S. mansoni* infection among school children of the study area may show that the children may be a potential source of infection and transmission for the diseases in the study area. Since it is well documented that these diseases are known to lower cognitive ability of students, low efficiency and productivity in their education endeavors may also be expected.

Other remarkable feature that has been shown from this study was high numbers of the children were infected with more than one type of worms. For example, one-tenth of the study participants were infected by both *S. mansoni* and *A. lumbricoides*. Such co-infections of *S.mansoni* and *A. lumbricoides* could be attributed to the co-endemicity of the two species and also poor sanitation in the study area. The finding of the present study was similar to study conducted in Cote d’Ivoire [33]. Co-infection may affect nutritional status of the children because of the combined effect of the different parasites that can deprive the important nutrients of the children.

When we analyze the risk factors associated with STH and *S. mansoni* infections, similar to a study conducted at Zarima town, northwest Ethiopia, the present study showed that *S. mansoni* infection had statistically significant association with swimming habit of school children similar to the present study [31]. The similarity may be because that both study area in near to water bodies in which swimming is common habit in both areas.

The strength of this study is; standard Kato Katz technique to detect ova of the parasites, qualified medical laboratory technologists and standard questionnaire were used. However, the study design (cross sectional) and the need to adjust the odds ratios for the effect of clustering by schools can be the limitations of the study.

## Conclusion

The current prevalence is showing that STHs and *S.mansoni* are still a major health problem for the community. Even though there were few attempts made previously like community mobilization in the study area, it did not result in significant reduction in the diseases occurrence. Therefore, periodic and effective community mobilization, health education targeting behavioral change and in addition, well organized control strategy with de worming must be implemented so that significant reduction in prevalence of STHs and *S. mansoni* can be realized.

## Abbreviations

OR: Odds ratio; STHs: Soil transmitted helminths; SPSS: Statistical package for the social sciences; WHO: World Health Organization.

## Competing interests

The authors declare that they have no competing interests.

## Authors’ contributions

BM: initiation of the study, design, implementation, analysis and write-up. AA: implementation, analysis and write-up. AA: implementation. MT: design. MA: implementation. ZA: analysis and write-up. DW: analysis and write-up. AK: analysis and write-up. All authors read and approved the final manuscript.
